# Red Blood Cell Distribution Width as a Potential Valuable Survival Predictor in Hepatitis B Virus-related Hepatocellular Carcinoma

**DOI:** 10.7150/ijms.79619

**Published:** 2023-05-29

**Authors:** Maoqing Tan, Bang Liu, Ruolan You, Qiqi Huang, Liyan Lin, Danni Cai, Rong Yang, Dongliang Li, Huifang Huang

**Affiliations:** 1 Central Laboratory, Fujian Medical University Union Hospital, Fuzhou, Fujian, 350001, China.; 2 Department of Hepatobiliary Disease, Fuzong Clinical Medical College of Fujian Medical University (900TH Hospital of Joint Logistics Support Force), Fuzhou, Fujian, 350025, China.; 3 Follow-up Center of Union Hospital Affiliated to Fujian Medical University, Fuzhou, Fujian, 350001, China.

**Keywords:** Red blood cell distribution width, Prognosis, Nomogram, Hepatitis B virus-related hepatocellular carcinoma

## Abstract

**Objectives:** Red blood cell distribution width (RDW) is a widely used clinical parameter recently deployed in predicting various cancers. This study aimed to evaluate the prognostic value of RDW in patients with hepatitis B virus (HBV)-related hepatocellular carcinoma (HCC).

**Methods:** We conducted a retrospective study of 745 patients with HBV-related HCC, 253 patients with chronic hepatitis B (CHB), and 256 healthy individuals to compare their hematological parameters and analyze their RDW levels. Potential risk factors for long-term all-cause mortality in patients with HBV-related HCC were predicted using Multivariate Cox regression. A nomogram was generated, and its performance was evaluated.

**Results:** The RDW of patients with HBV-related HCC was significantly higher than that of those with CHB and healthy controls. In the former, splenomegaly, liver cirrhosis, larger tumor diameter, multiple tumor number, portal vein tumor thrombus, and lymphatic or distant metastasis were significantly increased, and the later the Child-Pugh grade and Barcelona clinic liver cancer stage, the higher the RDW. Furthermore, multivariate Cox regression analysis identified RDW as an independent risk factor for predicting long-term all-cause mortality in patients with HBV-related HCC. Finally, we successfully generated a nomogram incorporating RDW and validated its predictive ability.

**Conclusions:** RDW is a potentially valuable hematological marker for predicting the survival and prognosis of patients with HBV-related HCC. The nomogram incorporating RDW can be used as an effective tool to plan the individualized treatment of such patients.

## Introduction

According to the Global Cancer Statistics 2020 study, primary liver cancer had an estimated 906,000 new cases and 830,000 deaths, making it the sixth most diagnosed cancer and the third most common cause of cancer mortality globally. Hepatocellular carcinoma (HCC) (75%-85%), intrahepatic cholangiocarcinoma (10%-15%), and other uncommon forms are among the primary liver cancers 1. Chronic hepatitis B virus (HBV) infection, which causes approximately 80% of all liver cancers in China, is the key risk factor for HCC 2. The Barcelona clinic liver cancer (BCLC) staging system has been extensively used for the past 20 years. It has become one of the most significant clinical models for providing accurate treatment plans and predicting the prognosis of patients with HCC 3, 4. However, owing to the high heterogeneity of patients, those in the same grading stage may have different prognoses. The prognosis for HCC remains poor, with a 5-year survival rate of only approximately 18%, despite the wide availability of modern therapy 5, 6. Therefore, discovering other complementary biomarkers or clinical decision models for identifying high-risk patients remains crucial.

A hematological characteristic called red blood cell distribution width (RDW), which indicates the heterogeneity of red blood cell volume, is frequently employed in the differential diagnosis of anemia 7, 8. Although traditionally used to assess anemia, elevated RDW is strongly associated with ineffective erythropoiesis, cardiovascular disease, venous thromboembolism, diabetes, kidney disease, liver disease, and various inflammatory and malignant diseases 9-15. In a propensity score matching study, Cheng et al. 16 proposed that in patients with stage I-II colorectal cancer, RDW is a poor independent predictor of disease-free survival (DFS), overall survival (OS), and cancer-specific survival (CSS). A retrospective analysis of the clinical data of 809 patients with laryngeal squamous cell carcinoma (LSCC) by Hsueh et al. 17 revealed that elevated RDW was linked to the poor OS, CSS, and DFS in patients with LSCC and that high levels of RDW were substantially associated with more mortality events. Pre- and post-treatment, as well as persistently high RDW, were demonstrated to be independently related to OS and CSS in Cheng et al.'s 18 analysis of 348 patients with prostate cancer. Yazici et al. 19 reported that individuals with higher RDW levels had a greater incidence of advanced cancer and were closely connected to short-term death in a study of patients with gastric cancer undergoing curative surgery. Similarly, Wan et al. 20 retrospectively analyzed the clinical information of 179 patients with esophageal cancer and found that preoperative RDW elevation independently predicted poorer DFS and OS in those patients.

Smirne et al. 6 reported that RDW might be a predictor of early death in patients with HCC; however, this was a relatively small study, and multiple etiologies of HCC were included, and RDW may vary depending on the different etiologies. Furthermore, in this study, the patients with HCC had mainly hepatitis C- and alcoholic liver disease-related HCC. In contrast, patients with HBV-related HCC accounted for only 12%, which could not fully reflect the prognostic value of RDW in these patients. Therefore, our study aimed to further elucidate the prognostic value of such patients and assess a nomogram model's predictive ability and clinical application by incorporating RDW into their survival prognosis.

## Materials and methods

### Participants

The cases of 745 patients with HBV-related HCC and 253 with chronic hepatitis B (CHB) who were first diagnosed at the 900TH Hospital of Joint Logistics Support Force (from January 2016 to December 2021) were evaluated in this study.

The following criteria were used to select patients with HBV-related HCC: (1) computed tomography (CT)/magnetic resonance imaging (MRI) or pathological diagnosis of HCC, (2) hepatitis B surface antigen positivity for >6 months, (3) diagnosis for the first time and no prior antitumor therapy, and (4) complete clinical and follow-up records. In addition, the exclusion criteria included the following: (1) superinfection with other hepatitis viruses; (2) combination with other etiologies, such as the use of alcohol or drugs or autoimmune hepatitis; (3) history of other cancers; (4) gastrointestinal bleeding over the past 6 months; (5) combination with other hematological diseases; and (6) combination with pregnancy or severe immune system deficiency.

The following criteria were used to select patients with CHB: (1) diagnosis per Chinese recommendations for the treatment and prevention of CHB (2019 version) 21 and (2) complete clinical and follow-up records. The following were the exclusion criteria: (1) liver cirrhosis, (2) portal hypertension, and (3) superinfection with other hepatitis viruses.

Additionally, 256 individuals without a history of cardiovascular or hematological illnesses and with normal liver and kidney function were enlisted as controls.

Ethical approval was obtained from the 900TH Hospital of Joint Logistics Support Force (approval number: 2022-014). The need for informed consent was waived for this investigation because all patient data were de-identified and anonymized before analysis.

### Demographic and clinical information

We gathered baseline demographic information (age, sex, and time of diagnosis), hematological parameters (complete blood cell count, coagulation function tests, serum biochemistry, and serum alpha-fetoprotein), and tumor information (tumor size, tumor number, existence of portal vein tumor thrombus [PVTT], and lymphatic or distant metastases). In addition, we recorded whether patients with HBV-related HCC had enlarged spleens or hepatocirrhosis. The Child-Pugh grade was calculated to describe liver function. Tumor staging at diagnosis used the BCLC criteria and was classified into five categories (0, A, B, C, and D). Prior to antitumor treatment, each indicator, including RDW, was detected.

### Definitions

In patients with HBV-related HCC, the diagnosis date was the date of the radiological diagnosis, not the date of the subsequent pathological confirmation. OS was also described as the period from the radiological diagnosis to the last follow-up or death resulting from any reason. Professional radiologists evaluated diagnostic CT scans or MRI results to determine the radiological tumor characteristics in patients with HBV-related HCC.

### Statistical analysis

Quantitative variables with non-normal distributions were presented as medians with interquartile ranges (25th-75th percentiles), whereas categorical variables were presented as absolute counts and percentages. A two-tailed chi-squared or Fisher's exact test was used to compare categorical variables. The Mann-Whitney rank-sum or Wilcoxon rank-sum test was used to compare non-normal continuous variables. Survival curves were calculated using the Kaplan-Meier method and compared using the log-rank test. Multiple Cox regression analysis with a forward likelihood ratio method was conducted to analyze the effect of multiple covariates on survival. In this study, we incorporated eight continuous variables (age at diagnosis, white blood cell count, red blood cell count, RDW, hematocrit, mean corpuscular volume, platelets, and maximum tumor diameter) and nine categorical variables (sex, Child-Pugh grade, alpha-fetoprotein, hepatocirrhosis, tumor number, PVTT, lymphatic metastasis, distant metastasis, and BCLC stage), and only the variables having a *p* < 0.05 were finally included into the model. Next, all patients with HBV-related HCC were randomly assigned to a training cohort (80%) or a validation cohort (20%) using R software. Subsequently, we developed a nomogram to determine the prognosis of patients with HBV-related HCC. The performance of the nomogram was evaluated through internal validation and calibration curves. After that, we assessed the nomogram's clinical value using the decision curve analysis (DCA). Finally, a total risk score (NomoScore: nomogram risk score) was calculated for each patient based on the nomogram models, and the cut-off point was automatically calculated using the X-tile software (Yale University, New Haven, CT, USA) to categorize the patients into the following three risk groups: high-, moderate-, and low-risk.

The R software 4.1.3 (RStudio Inc., Boston, MA, USA) and SPSS 26.0 (IBM, Armonk, NY, USA) were used for statistical analysis, and Adobe Illustrator (Adobe Systems, San Jose, CA, USA) and GraphPad Prime 9.0 (GraphPad, La Jolla, CA, USA) were used for plotting. Statistics were deemed to be significant for any value with *p* < 0.05.

## Results

### Baseline characteristics of enrolled participants

Table 1 (Figure S1) in this study lists the participants' clinical and demographic characteristics. The findings revealed that the RDW value was the highest in patients with HBV-related HCC (13.80%), followed by CHB patients (13.30%), and lowest in healthy controls (12.90%), with *p* values < 0.05.

### Subgroup analysis of the patients with HBV-related HCC

We subsequently assessed the differences in RDW levels in each subgroup in the total cohort of patients with HBV-related HCC, as reported in Table S1 and Figure S2, which did not differ based on age or sex. However, when patients with HBV-related HCC had an enlarged spleen or liver cirrhosis, their RDW levels were significantly increased (enlarged spleen vs. normal-sized spleen: 14.3% vs. 13.6%; cirrhosis vs. non-cirrhosis: 13.9% vs. 13.6%), and the higher the Child-Pugh grade, the more significant the increase in RDW (RDW of grades A, B, and C were 13.60%, 15.20%, and 17.00%, respectively). In addition, the level of RDW also increased significantly when the maximum diameter of the tumor was >10 cm, the number of tumors was >1, or patients had PVTT, lymphatic metastasis, or distant metastasis. Furthermore, patients with advanced BCLC stages had RDW values much significantly higher than those with early stages—for stages 0-D, the RDW values were 13.50%, 13.50%, 13.70%, 14.20%, and 16.60%, respectively. Moreover, we found that in the 'death' group, the RDW level was significantly higher than that in the 'alive' group (14.20% vs. 13.50%), with all* p* values < 0.001.

### Multivariate Cox regression analysis

Eight variables persisted in the model after the Cox proportional hazard regression models were utilized to determine independent prognostic factors for OS (Supplementary Table 2). The results revealed that age (HR 1.012, 95% CI 1.003-1.022), RDW (HR 1.078, 95% CI 1.014-1.146), Child-Pugh grade (grade B [HR 2.008, 95% CI 1.542-2.614], grade C [HR 6.203, 95% CI 2.541-15.147], regarding grade A), maximum tumor diameter (HR 1.059, 95% CI 1.034-1.085), number of tumors (HR 1.892, 95% CI 1.421-2.519), PVTT (HR 1.526, 95% CI 1.029-2.263), distant metastasis (HR 1.570, 95% CI 1.146-2.151), and BCLC stage (stage B [HR 3.28, 95% CI 1.404-7.66], stage C [HR 6.04, 95% CI 2.523-14.38], stage D [HR 6.85, 95% CI 2.110-22.29], regarding stage 0) were independent risk factors for long-term all-cause mortality in patients with HBV-related HCC, with all *p* < 0.05.

### Construction and validation of the prognosis nomogram

**Figure 1:**
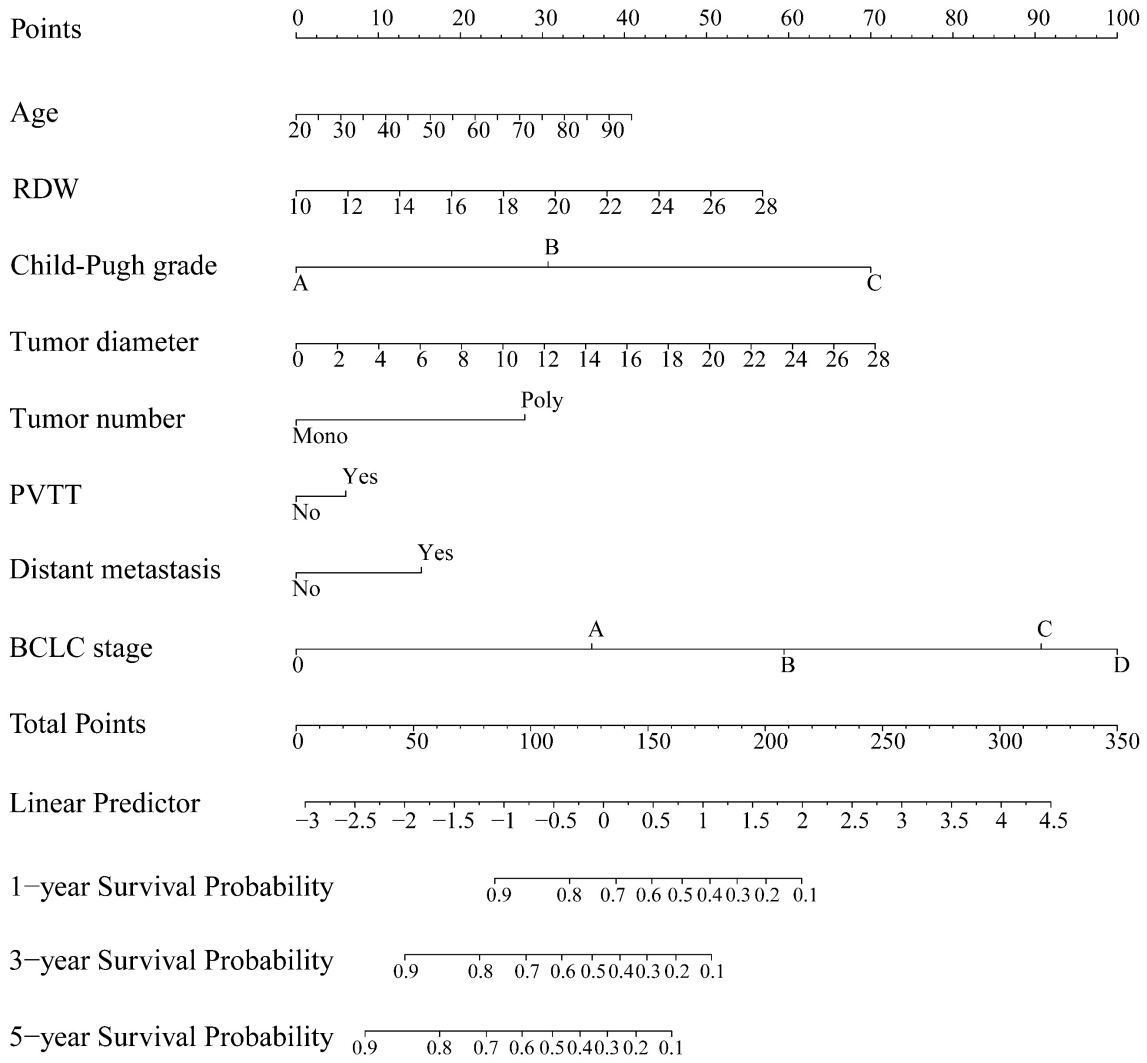
Survival nomogram for patients with HBV-related HCC. HBV-related HCC: hepatitis B virus-related hepatocellular carcinoma; RDW: Red blood cell distribution width; PVTT: portal vein tumor thrombus; BCLC: Barcelona clinic liver cancer.

**Figure 2:**
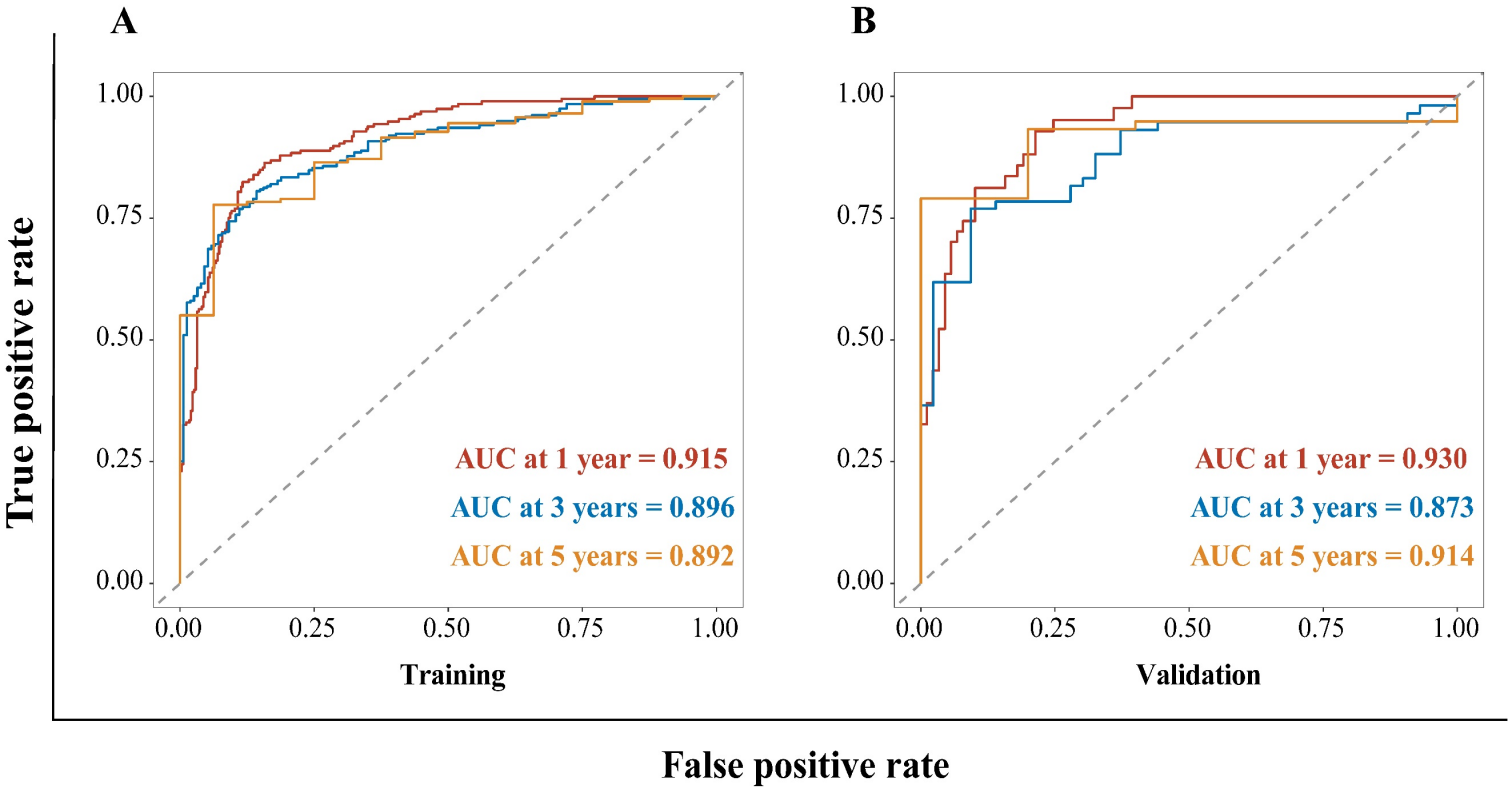
AUC for predicting the 1-, 3-, and 5-year OS in the training cohort (A) and validation cohort (B). AUC: area under the curve; OS: overall survival.

**Figure 3:**
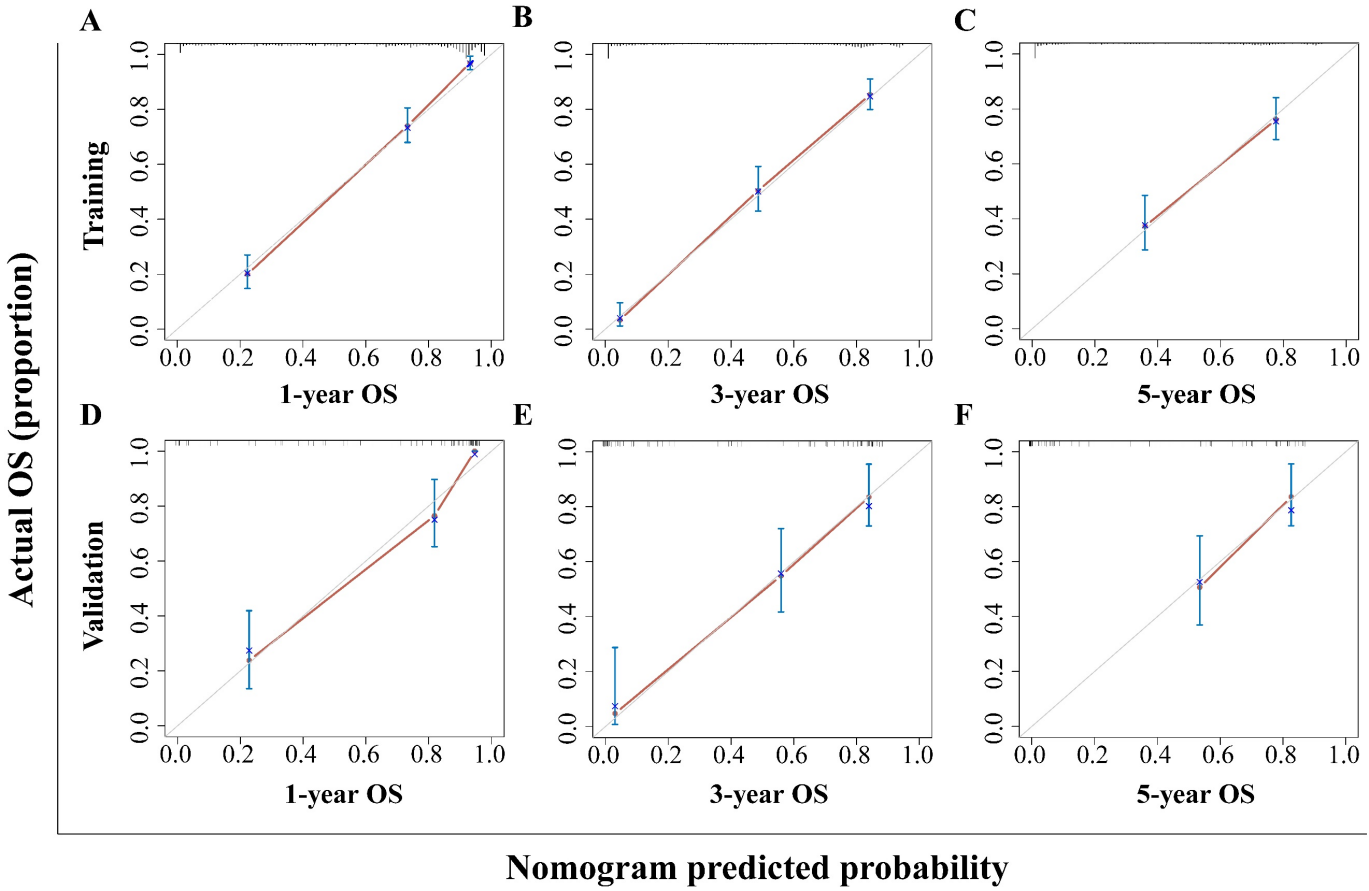
Calibration curve for the nomogram. The 1- (A), 3- (B), and 5- (C) year OS rates in the training cohort; the 1- (D), 3- (E), and 5- (F) year OS rates in the validation cohort. The x-axis represents nomogram-predicted survival; the y-axis represents actual survival. OS: overall survival.

The HBV-related HCC cohort was randomly divided into training and validation groups to develop a nomogram for individualized clinical treatment. The two groups of patients were not significantly different in terms of diverse basic variables, such as age, RDW, Child-Pugh grade, maximum tumor diameter, tumor number, PVTT, distant metastasis, and BCLC stage (*p* > 0.05, Table S3). In our predictive model (Figure 1)—the most important factor affecting patient survival was the BCLC stage, followed by the Child-Pugh grade. In the training cohort, 0.915, 0.896, and 0.892 corresponded to areas under the curve (AUCs) of 1-, 3-, and 5 years, respectively, while in the validation cohort, the corresponding AUCs were 0.930, 0.873, and 0.914, respectively (Figure 2), indicating that the model bore good discriminatory power. Furthermore, in the training and validation sets, the calibration curves for OS, namely 1-year OS, 3-year OS, and 5-year OS, were in good agreement with their standard lines (Figure 3), indicating that the model bore good accuracy.

**Figure 4:**
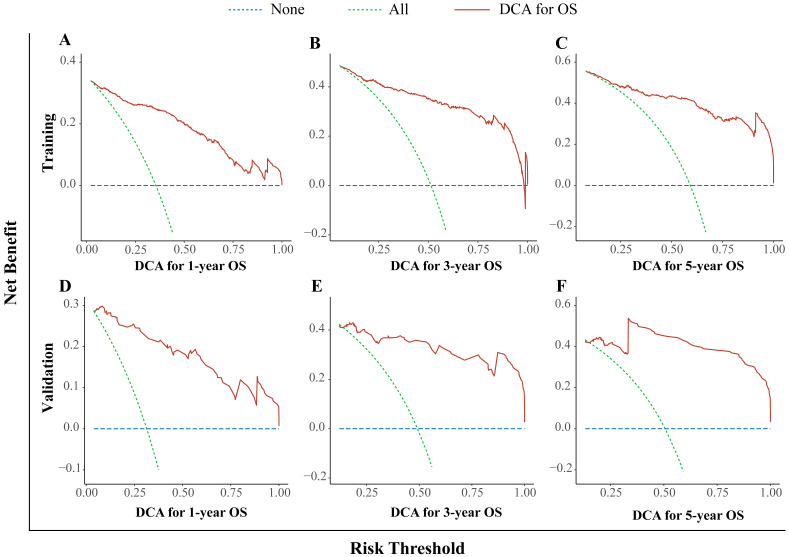
Decision curve analysis for the nomogram. The 1- (A), 3- (B), and 5- (C) year OS rates in the training cohort; the 1- (D), 3- (E), and 5- (F) year OS rates in the validation cohort. DCA: decision curve analysis; OS: overall survival.

In this study, we used the DCA curve to evaluate the net clinical benefits of the predictive models. The results demonstrated that the nomogram model constructed could significantly increase the net benefit in predicting the 1-, 3- and 5-year OS rates, and it showed a wide range of threshold probabilities (Figure. 4).

### Stratification of survival risk and assessment of prognosis

**Figure 5:**
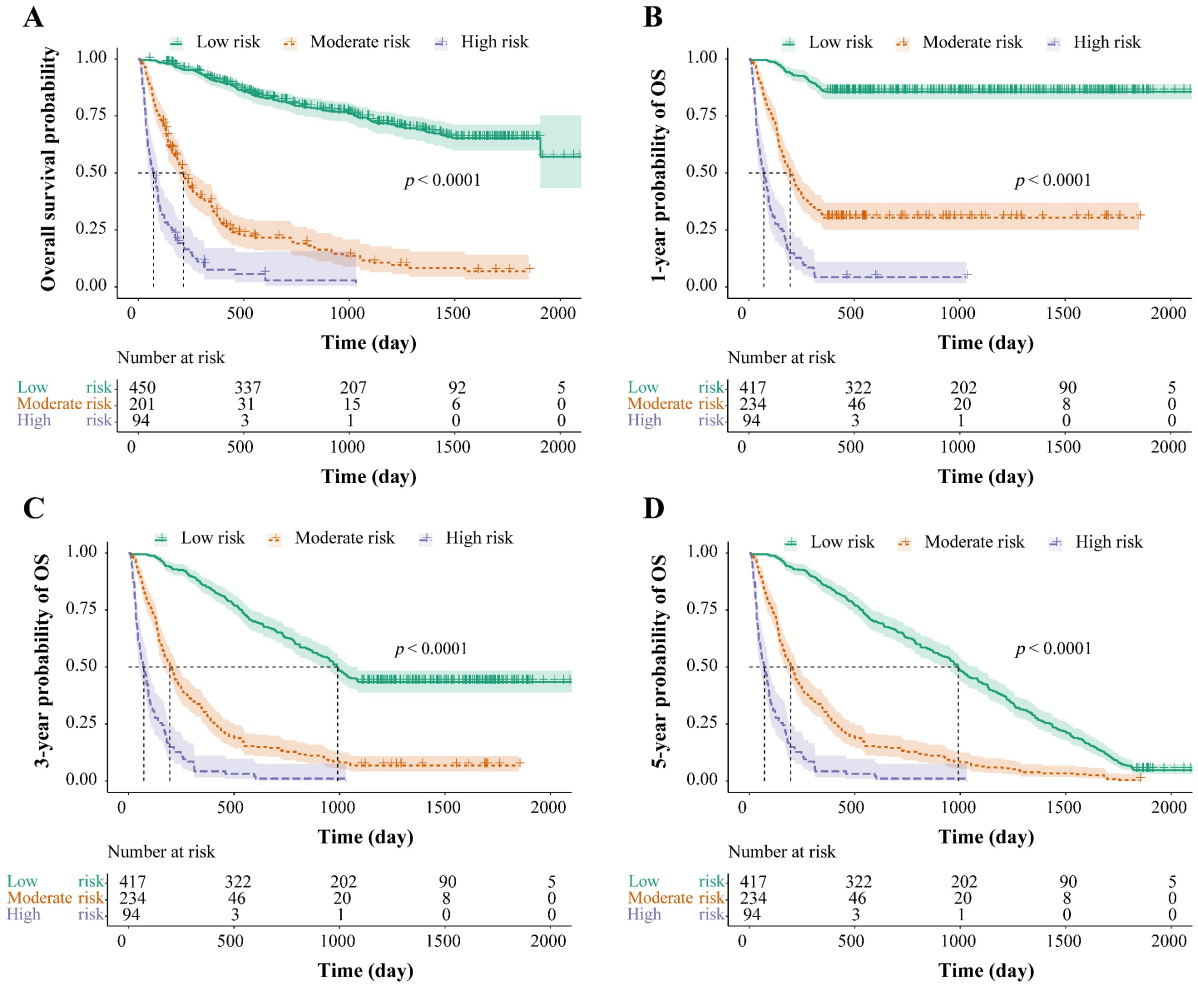
Risk stratification for survival, based on the nomogram risk scores. Risk stratification for OS (A), risk stratification for 1-year OS (B), risk stratification for 3-year OS (C), and risk stratification for 5-year OS (D). OS: overall survival.

The total risk NomoScores were calculated for each patient according to the nomogram model, and the patients with HBV-related HCC were categorized into different risk groups using the optimal cut-off points to further judge the capability of the nomogram to discriminate the survival. In the OS group, the total risk NomoScores of > 208 was the high-risk group, < 151 was the low-risk group, and those in between were the moderate-risk group. In the 1-, 3-, and 5-year OS groups, the high-risk group was defined as a total risk NomoScores > 208, the low-risk < 142, and the rest fell into the moderate-risk group. Finally, the survival curves were calculated using the Kaplan-Meier method, and the OS, 1-, 3-, and 5-year OS of the three different risk groups were compared. The results showed that risk grouping based on the NomoScores had a significant value in identifying mortality (*p* < 0.05, Figure. 5).

## Discussion

This study appears to be the largest study to illustrate the prognostic value of RDW in patients with HBV-related HCC. Our study demonstrates that elevated RDW is an independent, unfavorable predictor of mortality in individuals with HBV-related HCC. The nomogram we constructed has high clinical application value and can assist clinicians in formulating more appropriate diagnosis and treatment plans and devising patients' prognoses.

According to our results, patients with CHB bore higher RDW than healthy controls, which is consistent with other descriptions 22. Based on this, we also discovered that patients with HBV-related HCC had the highest RDW levels among all participants. Subsequently, in a subgroup analysis of patients with HBV-related HCC, we discovered that RDW was considerably higher in patients with cirrhosis, which is consistent with earlier research on the relationship between RDW and HBV-related liver cirrhosis 23. Aside from that, we noticed that RDW was strongly linked to greater tumor diameter, more tumors overall, PVTT, lymphatic metastasis, or distant metastasis. Similarly, RDW values increased in line with patients' higher Child-Pugh grades, advanced BCLC stages, or late-death status. Therefore, we can deduce that RDW may be linked to a poorer prognosis for patients with HBV-related HCC and may potentially be a predictor of these patients' survival prognosis. Notably, the baseline age was not completely balanced among the three groups of participants, which the patients with HBV-related HCC had a highest median age, while those with CHB had a lowest median age. To exclude the influence of age on RDW, a correlation analysis was performed, and the results showed no significant correlation between age and RDW in these three groups (*p* > 0.05, Figure S3). In addition, after adjusting for age in the multivariate Cox regression analysis, RDW remains an independent risk factor for long-term all-cause mortality in patients with HBV-related HCC.

Nomograms can simplify and enable the visualization of complex regression equations, making the results of predictive models easier to understand. Therefore, we attempted to construct a nomogram for individualized clinical treatment. To the best of our knowledge, this is the first time that RDW, an inexpensive and readily available marker, has been incorporated into a prognostic model for patients with HBV-related HCC. Subsequently, we analyzed the AUC, calibration curve, DCA curve, and risk stratification of the prediction model for 1-, 3-, and 5-year OS. Overall, the results indicated that our nomogram has relatively high discrimination and accuracy for predicting OS in patients with HBV-related HCC, which could significantly increase the net benefit in predicting survival and showed a good clinical application value.

It is well known that, in a living organism with blood, the hematological system interacts with and influences tissues and organs continuously, constantly exchanging substances and energy, such as respiration gases, metabolites, lipids, and sugars. Due to the pronounced fluidity of blood and its window- and mirror-like characteristics, the hematological system not only reflects the many physiological and pathological conditions of the human body but also serves as a link and “messenger” for interaction and information transmission in the maintenance, or imbalance, of homeostasis between tissues and organs. Changes in blood cell parameters reflect not only the status of the hematopoietic system but also the functional changes in other systems. RDW has been suggested as an integrative biomarker for multidimensional dysfunctional physiology 24, revealing its changes in different disease states and how these changes affect the occurrence and development of disease will provide a wider theoretical framework for precisely assessing the likelihood of a specific disease prognosis.

Inflammation, malnutrition, and oxidative stress are examples of putative pathways that could explain the link between RDW and a poor prognosis in patients with HBV-related HCC. First, inflammation is one of the major underlying factors in the onset and progression of cancer 25. Given the significant role RDW plays in inflammation, the association between RDW and cancer has garnered much interest. RDW levels have been linked to inflammatory markers like interleukin-6, tumor necrosis factor α, and C-reactive protein in many malignancies and inflammatory disorders, according to earlier research 26, 27. On the other hand, inflammation impacts erythropoiesis and death through direct myelosuppression of erythroid precursors, decreased synthesis of erythropoietin, decreased iron bioavailability, and promoted erythrocyte apoptosis 28. However, a more advanced tumor stage may secrete more cytokines and release more tumor-degradation products, leading to a greater degree of systemic inflammation 29. This, in turn, enhances the inhibitory influence on erythropoiesis and accelerates the apoptosis of erythrocytes, shortening the erythrocyte lifespan and raising RDW levels. Second, long-term chronic consumption and decreased appetite in patients with advanced cancer frequently result in deficiencies in many trace elements and vitamins, including iron, folic acid, and vitamin B_12_, which in turn causes an increase in RDW. Third, recent studies have suggested that oxidative stress plays a significant role in regulating the homeostasis of hematopoietic cells, especially erythrocytes and hematopoietic stem cells, which are highly sensitive to the deregulated accumulation of reactive oxygen species (ROS). When ROS accumulation is out of control, red blood cells tend to be destroyed, and their lifespan is shortened 30, leading to elevated RDW levels. Therefore, high RDW levels are well-suited to reflect both ongoing chronic inflammation and poor nutritional status in patients with HBV-related HCC. However, further research is necessary to determine the precise mechanism underlying its link to a poor prognosis in patients with HBV-related HCC.

In our series of studies, we discovered that individuals with HBV-related HCC who had elevated RDW had a poor prognosis. Therefore, we suggested that RDW might be used to improve these patients' prognoses. Nevertheless, this study bears some limitations. First, there may have been selection bias due to its retrospective nature. Second, the effect of age on RDW cannot be completely denied. Third, the variables included in the study were insufficiently broad; for instance, the lack of clinical data availability prevented the inclusion of HBV-DNA. Finally, we did not dynamically monitor changes but merely used a single pretreatment RDW value as a prognostic predictor. Therefore, high-quality prospective randomized controlled trials are needed to confirm the prognostic significance of RDW in patients with HBV-related HCC.

## Conclusion

RDW, a simple and widely used hematological parameter, is significantly increased in patients with HBV-related HCC and is associated with poor prognosis, which may be utilized as a potential prognostic indicator for those patients. In addition, the nomogram generated by incorporating RDW has high accuracy in predicting the survival and prognosis of patients with HBV-related HCC and can be used as an effective tool for individualized clinical treatment and prognosis judgment.

## Supplementary Material

Supplementary figures and tables.

## Figures and Tables

**Table 1:** Demographic and clinical characteristics of the participants

	Healthy control (n = 256)	CHB (n = 253)	HBV-related HCC (n = 745)
Age (years)	41 (29, 54)	37 (30, 47) ^c^	55 (47, 64) ^a b^
Sex (M/F)	176/80	196/57	638/107
ALT (U/L)	15.25 (11.70, 22.58)	417.00 (193.90, 840.85) ^c^	37.30 (25.10, 65.65) ^a b^
AST (U/L)	16.85 (14.15, 19.90)	204.80 (95.05, 458.45) ^c^	46.50 (28.20, 95.40) ^a b^
T-bil (μmol/L)	10.00 (7.13, 13.35)	21.90 (13.70, 41.95) ^c^	15.30 (11.15, 24.05)^ a b^
Alb (g/L)	44.40 (41.93, 46.68)	41.10 (37.20, 44.60) ^c^	40.30 (36.20, 44.40) ^a^
PT (S)	11.20 (10.80, 11.70)	12.20 (11.50, 13.50) ^c^	12.40 (11.70, 13.40)^ a^
INR	0.98 (0.94, 1.02)	1.07 (1.00, 1.19) ^c^	1.08 (1.02, 1.18) a
ALP (U/L)	64.00 (54.00, 77.93)	101.00 (76.70, 127.50) ^c^	105.00 (78.75, 158.75) ^a^
GGT (U/L)	18.65 (13.55, 26.98)	118.80 (70.75, 186.40) ^c^	99.00 (44.00, 218.50) ^a^
WBC (× 10^9^/L)	6.18 (5.24, 7.14)	5.61 (4.66, 6.62) ^c^	5.65 (4.56, 7.35) ^a^
NEU (× 10^9^/L)	3.36 (2.72, 4.27)	2.90 (2.37, 3.55) ^c^	3.47 (2.63, 4.75) ^b^
LYM (× 10^9^/L)	2.02 (1.67, 2.41)	1.81 (1.50, 2.45) ^c^	1.44 (1.06, 1.89) ^a b^
RBC (× 10^12^/L)	4.67 (4.40, 4.96)	4.59 (4.25, 4.92)	4.51 (3.99, 4.93) ^a^
HCT (%)	42.40 (39.80, 45.00)	42.70 (39.35, 45.20)	40.90 (37.00, 44.30) ^a b^
MCV (fL)	90.30 (87.90, 93.58)	92.80 (89.90, 95.80) ^c^	92.30 (87.40, 96.10) ^a^
RDW (%)	12.90 (12.50, 13.40)	13.30 (12.60, 14.30) ^c^	13.80 (13.00, 14.90) ^a b^
PLT (× 10^9^/L)	222.50 (195.00, 254.75)	176.00 (136.50, 205.00) ^c^	169.00 (117.50, 225.00) ^a^

Data were expressed as the medians (IQR) or n (%). ^a^*p* < 0.05 When HBV-related HCC and healthy controls were compared. ^b^*p* < 0.05 When HBV-related HCC and CHB were compared. ^c^*p* < 0.05 When CHB and healthy controls were compared. ALT: alanine aminotransferase; AST: aspartate aminotransferase; T-bil: total bilirubin; Alb: albumin; PT: prothrombin time; INR: International Normalized Ratio; ALP: alkaline phosphatase; GGT: γ-glutamyl transpeptidase; WBC: white blood cell; NEU: neutrophil; LYM: lymphocyte; RBC: red blood cell; HCT: hematocrit; MCV: mean corpuscular volume; RDW: Red blood cell distribution width; PLT: platelet.

## Abbreviations

HCC: hepatocellular carcinoma
HBV: hepatitis B virus
BCLC: Barcelona clinic liver cancer
RDW: red blood cell distribution width
DFS: disease-free survival
OS: overall survival
CSS: cancer-specific survival
LSCC: laryngeal squamous cell carcinoma
CHB: chronic hepatitis B
PVTT: portal vein tumor thrombus
DCA: decision curve analysis
AUC: areas under the curve
ROS: reactive oxygen species
