# Supplementary material for: Red Blood Cell Distribution Width as a Potential Valuable Survival Predictor in Hepatitis B Virus-related Hepatocellular Carcinoma
Source: Int J Med Sci. 2023 May 29;20(7):976–84. doi: 10.7150/ijms.79619 (PMC10266047; doi:10.7150/ijms.79619)
Supplement: Supplementary file 1 — Supplementary figures and tables. [file ijmsv20p0976s1.pdf]

Table S1

Subgroup analyses for RDW in the HBV-related HCC patients.

	RDW (%)	*p* value
Age (n)		0.857
≤ 55 (379)	13.70 (13.00, 14.90)	
> 55 (366)	13.80 (13.10, 15.00)	
Sex (n)		0.537
Female（107）	13.70 (13.00, 14.40)	
Male（638）	13.80 (13.00, 14.93)	
Child-Pugh grade (n)		< 0.001
A (584)	13.60 (12.90, 14.30)	
B (134)	15.20 (14.10, 16.23)	
C (27)	17.00 (15.50, 18.90)	
Enlarged spleen (n)		< 0.001
Yes (318)	14.30 (13.40, 15.60)	
No (427)	13.60 (12.90, 14.30)	
Hepatocirrhosis (n)		< 0.001
Yes (507)	13.90 (13.10, 15.20)	
No (238)	13.60 (13.00, 14.30)	
Tumor diameter (n)		< 0.001
≤ 2 (97)	13.50 (12.85, 14.40)	
> 2, ≤ 5 (216)	13.60 (12.90, 14.58)	

> 5, ≤ 10 (219)	13.70 (12.90, 14.70)	
> 10 (213)	14.30 (13.50, 16.10)	
Tumor number (n)		< 0.001
Mono	13.60 (12.90, 14.50)	
Poly	14.10 (13.20, 15.40)	
PVTT (n)		< 0.001
Yes (244)	14.40 (13.40, 15.90)	
No (501)	13.60 (12.90, 14.45)	
Lymphatic metastasis (n)		< 0.001
Yes (82)	14.45 (13.60, 16.00)	
No (663)	13.70 (13.00, 14.80)	
Distant metastasis (n)		< 0.001
Yes (71)	14.60 (13.70, 15.90)	
No (674)	13.70 (13.00, 14.80)	
BCLC stage(n)		< 0.001
0 (76)	13.50 (12.83, 14.40)	
A (268)	13.50 (12.80, 14.30)	
B (113)	13.70 (13.00, 14.55)	
C (255)	14.20 (13.20, 15.40)	
D (33)	16.60 (15.15, 17.65)	
Death (n)		< 0.001
Yes (361)	14.20 (13.30, 15.60)	

No (384)	13.50 (12.90, 14.30)

PVTT: portal vein tumor thrombus; BCLC: Barcelona clinic liver cancer.

Table S2

Multivariate Cox regression analysis with a forward likelihood ratio method for OS of HBV-related HCC patients.

	B	SE	Wald	*p* value	HR	95% CI
Age	0.012	0.005	6.387	0.011	1.012	1.003-1.022
RDW	0.075	0.031	5.742	0.017	1.078	1.014-1.146
Child-Pugh grade			34.789			
A					1.000	
B	0.697	0.135	26.801	<0.001	2.008	1.542-2.614
C	1.825	0.455	16.056	<0.001	6.203	2.541-15.147
Tumour diameter	0.058	0.012	22.183	<0.001	1.059	1.034-1.085
Tumour number						
Mono					1.000	
Poly	0.638	0.146	19.057	<0.001	1.892	1.421-2.519
PVTT						
No					1.000	
Yes	0.423	0.201	4.426	0.035	1.526	1.029-2.263
Distant metastasis						
No					1.000	
Yes	0.451	0.161	7.871	0.005	1.570	1.146-2.151
BCLC stage			24.471			
0					1.000	

A	0.711	0.403	3.117	0.077	2.037	0.925-4.487
B	1.188	0.433	7.524	0.006	3.280	1.404-7.664
C	1.799	0.446	16.293	<0.001	6.045	2.523-14.382
D	1.926	0.601	10.250	0.001	6.859	2.110-22.294

RDW: red blood cell distribution width; PVTT: portal vein tumor thrombus; BCLC: Barcelona clinic liver cancer.

Table S3

Comparison of basic variables after randomly assigned between training and validation cohorts.

Variables	Training cohorts (n = 596)	Validation cohorts (n = 149)	*p* value
Age	55 (47, 64)	55 (45, 63)	0.779
RDW	13.80 (13.03, 14.90)	13.70 (13.00, 15.00)	0.543
Child-Pugh grade			0.203
A	469 (78.69%)	115 (77.18%)	
B	109 (18.29%)	25 (16.78%)	
C	18 (3.02%)	9 (6.04%)	
Tumour diameter	5.95 (3.00, 10.40)	6.40 (3.20, 11.50)	0.251
Tumor number			0.712
Mono	330 (55.37%)	85 (57.05%)	
Poly	266 (44.63%)	64 (42.95%)	
PVTT			0.668
No	403 (67.62%)	98 (65.77%)	
Yes	193 (32.38%)	51 (34.23%)	
Distant metastasis			0.318
No	536 (89.93%)	138 (92.62%)	
Yes	60 (10.07%)	11 (7.38%)	
BCLC stage			0.861

0	64 (10.74%)	12 (8.05%)
A	213 (35.74%)	55 (36.91%)
B	91 (15.27%)	22 (14.77%)
C	203 (34.06%)	52 (34.90%)
D	25 (4.19%)	8 (5.37%)

RDW: red blood cell distribution width; PVTT: portal vein tumor thrombus; BCLC: Barcelona clinic liver cancer.

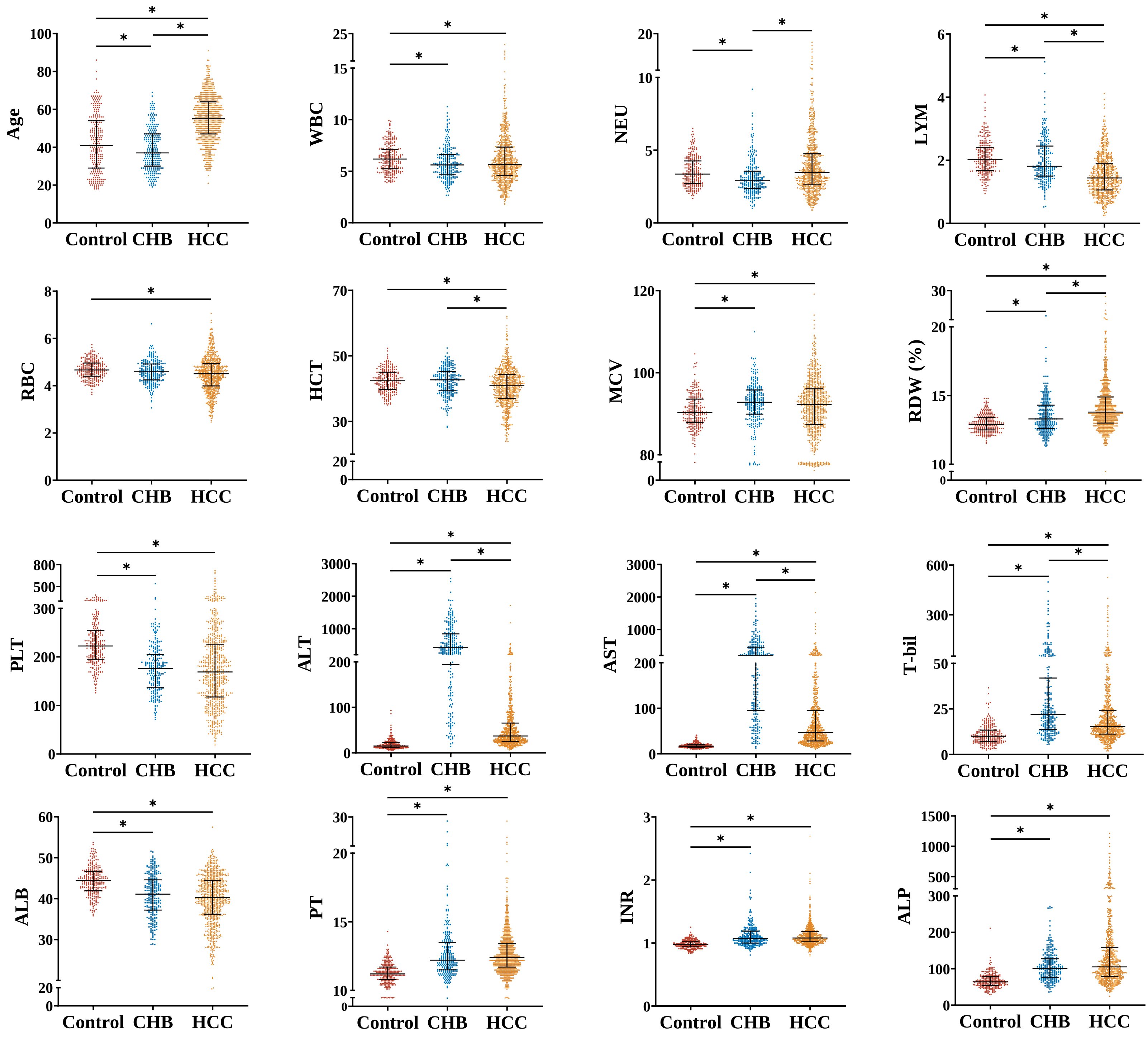
Age
WBC
NEU
LYM
RBC
HCT
MCV
RDW (%)
PLT
ALT
AST
T-bil
ALB
PT
INR
ALP
Control
CHB
HCC
*

Figure S1. Demographic and clinical characteristics of the subjects.

Control: healthy control; CHB: chronic hepatitis B; HBV-related HCC: hepatitis B virus-related hepatocellular carcinoma; WBC: white blood cell; NEU: neutrophil; LYM: lymphocyte; RBC: red blood cell; HCT: hematocrit; MCV: mean corpuscular volume; RDW: Red blood cell distribution width; PLT: platelet; ALT: alanine aminotransferase; AST: aspartate aminotransferase; T-bil: total bilirubin; Alb: albumin; PT: prothrombin time; INR: International Normalized Ratio; ALP: alkaline phosphatase.

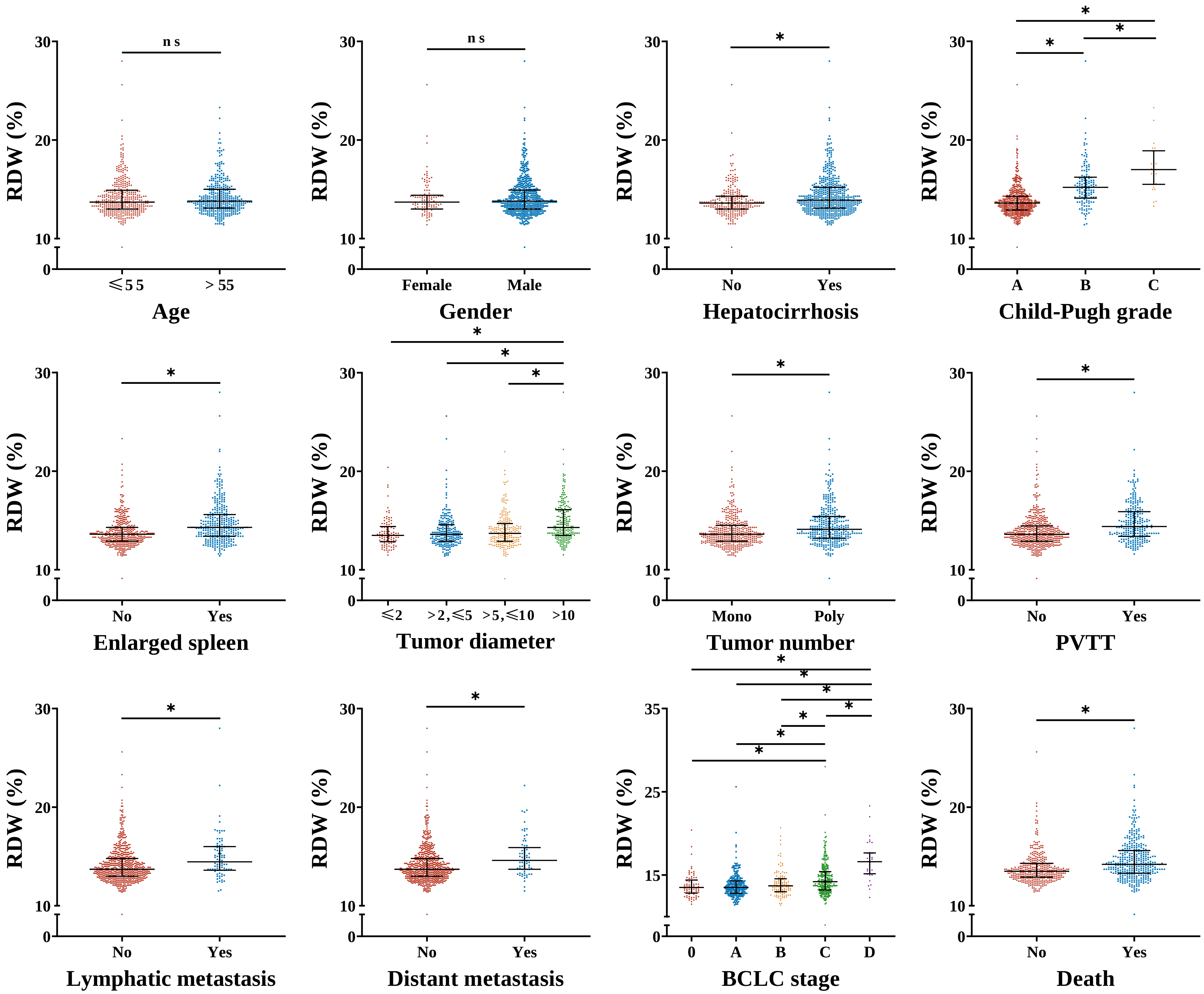
RDW (%)
n s
≤55
> 55
Age
n s
Female
Male
Gender
*
No
Yes
Hepatocirrhosis
*
A
B
C
Child-Pugh grade
*
No
Yes
Enlarged spleen
*
≤2
>2,≤5
>5,≤10
>10
Tumor diameter
*
Mono
Poly
Tumor number
*
No
Yes
PVTT
*
No
Yes
Lymphatic metastasis
*
No
Yes
Distant metastasis
*
0
A
B
C
D
BCLC stage
*
No
Yes
Death

Figure S2. Subgroup analyses for RDW in the patients with HBV-related HCC.

HBV-related HCC: hepatitis B virus-related hepatocellular carcinoma; RDW: red blood cell distribution width; PVTT: portal vein tumor thrombus; BCLC: Barcelona clinic liver cancer.

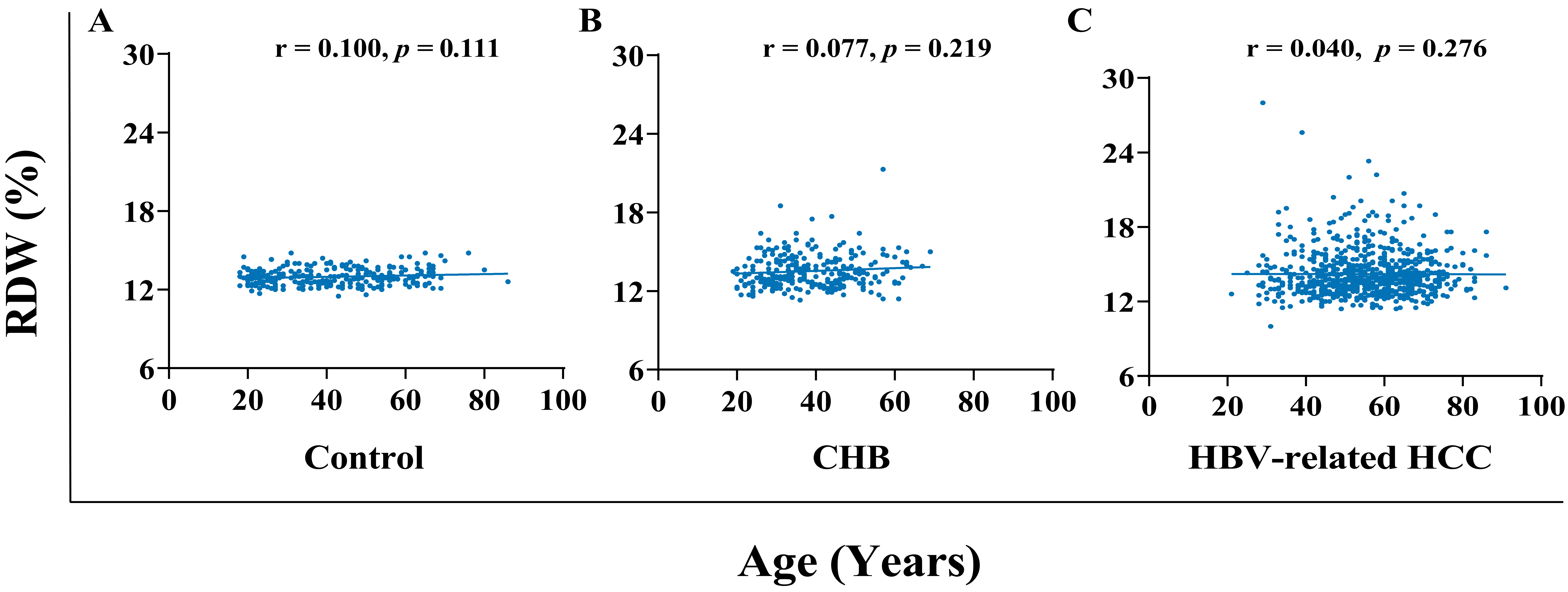
A
r = 0.100, p = 0.111
B
r = 0.077, p = 0.219
C
r = 0.040, p = 0.276
RDW (%)
30
24
18
12
6
0
20
40
60
80
100
Control
CHB
HBV-related HCC
Age (Years)

Figure S3. Correlation analysis between RDW and age in healthy control (A), patients with CHB (B) and HBV-related HCC (C).

Control: healthy control; CHB: chronic hepatitis B; HBV-related HCC: hepatitis B virus-related hepatocellular carcinoma; RDW: Red blood cell distribution width.
